# Impact of Age-related Diseases on Pulmonary Function Tests in Older Japanese Adults: A Cross-sectional Pilot Study

**DOI:** 10.31662/jmaj.2019-0076

**Published:** 2020-06-19

**Authors:** Mitsuhiro Matsuo

**Affiliations:** 1 Department of Anesthesiology, Itoigawa General Hospital, Itoigawa, Japan

**Keywords:** aging, frailty, reference value, spirometry

## Abstract

**Introduction:**

A widely used reference range for pulmonary function testing was derived from middle-aged, healthy, non-smoking adults in Japan. This study examined the effect of age-related diseases on pulmonary function tests for older Japanese adults.

**Methods:**

All patients aged ≥65 years who underwent spirometry before general and orthopedic surgeries in Itoigawa General Hospital (Niigata, Japan) from January 2014 to June 2019 were identified, and their charts were reviewed.

**Results:**

This study included 1050 Japanese patients (median age: 75 years). The median spirometric values of vital capacity, forced vital capacity (FVC), forced expiratory volume in 1 second (FEV1), and FEV1/FVC in all patients were 2.66 L [interquartile range; 2.24, 3.25], 2.57 L [2.13, 3.13], 1.98 L [1.66, 2.37], and 77.5% [72.2, 81.9], respectively. Multiple regression analyses revealed that spirometric values were significantly affected by age, body height, sex, smoking status, social dependency, dyslipidemia, diabetes, history of heart failure, peripheral artery disease, end-stage renal disease, neuromuscular disease, and psychiatric disorders. Male sex and height were positively correlated with FVC and FEV1. Other factors, such as a history of heart failure, neuromuscular disease, and independent physical activity, were negatively correlated with FVC and FEV1 to almost the same extent as that of age.

**Conclusions:**

These data will provide clinically useful information to accurately interpret pulmonary function test results in older Japanese adults.

## Introduction

Pulmonary function testing is considered as the basis for diagnosis in many categories of pulmonary disease ^(1)^. Aging causes a loss of functional reserves in many organ systems ^(2)^, and respiratory function declines in an age-dependent manner ^(3)^. Although there is an increasing number of older patients with respiratory diseases in Japan ^(4), (5)^, a widely used reference range for spirometry is mainly derived from middle-aged adults who are healthy non-smokers ^(3)^. Older patients often have many types of pre-existing comorbidities and geriatric syndrome. Diversity among age-related diseases should be considered in order to have sufficient understanding of the respiratory functions of older patients.

Pre-anesthetic and pre-surgical assessments include a complete medical history, physiological assessment, current medication, and social status ^(6)^. Pulmonary function tests were routinely conducted before general and orthopedic surgeries in the author’s hospital. This study examined the effect of age-related diseases on pulmonary function tests by secondary use of preoperative data evaluated before non-thoracic surgery for patients aged 65 years and older.

## Materials and Methods

### Study design

This cross-sectional study was approved by the ethics committee of Itoigawa General Hospital (#2019-11) and conducted in accordance with the principles of the Declaration of Helsinki. All patients aged ≥65 years who underwent spirometry before general and orthopedic surgeries in Itoigawa General Hospital from January 2014 to June 2019 were identified, and their charts were reviewed. Exclusion criteria included thoracic surgery; any known respiratory disease; chest X-ray abnormalities, such as infiltrations and pleural effusion; postpneumonectomy; and race other than Japanese. Vital capacity (VC), forced VC (FVC), and forced expiratory volume in 1 second (FEV1) were measured using the standard technique ^(7)^ with the HUDAC-77 (Fukuda Denshi Co., Ltd., Tokyo, Japan). Healthcare providers distributed questionnaires on patients’ smoking histories, including information on current smokers, former smokers who had not smoked for more than 1 month, or those who had never smoked. The answer for pack-years was optional. Based on the long-term care insurance (LTCI) law in Japan, the LTCI applicant is assigned to one of the levels of care required (certified support level of 1-2 or care level of 1-5 ^(2)^. Patients who were certified at the LTCI support level and who did not require this service were regarded as physically independent. Those who had been diagnosed with dementia were included. Patients who were receiving anti-hypertensive, lipid-lowering, and anti-diabetic drugs were regarded as having hypertension, dyslipidemia, and diabetes mellitus (DM), respectively. Liver cirrhosis with Child–Pugh class B or C was included. End-stage renal disease (ESRD) was defined as an estimated glomerular filtration rate of < 15 mL/min/1.73 m^2^ or requiring dialysis.

### Statistical analysis

**Figure 1.**
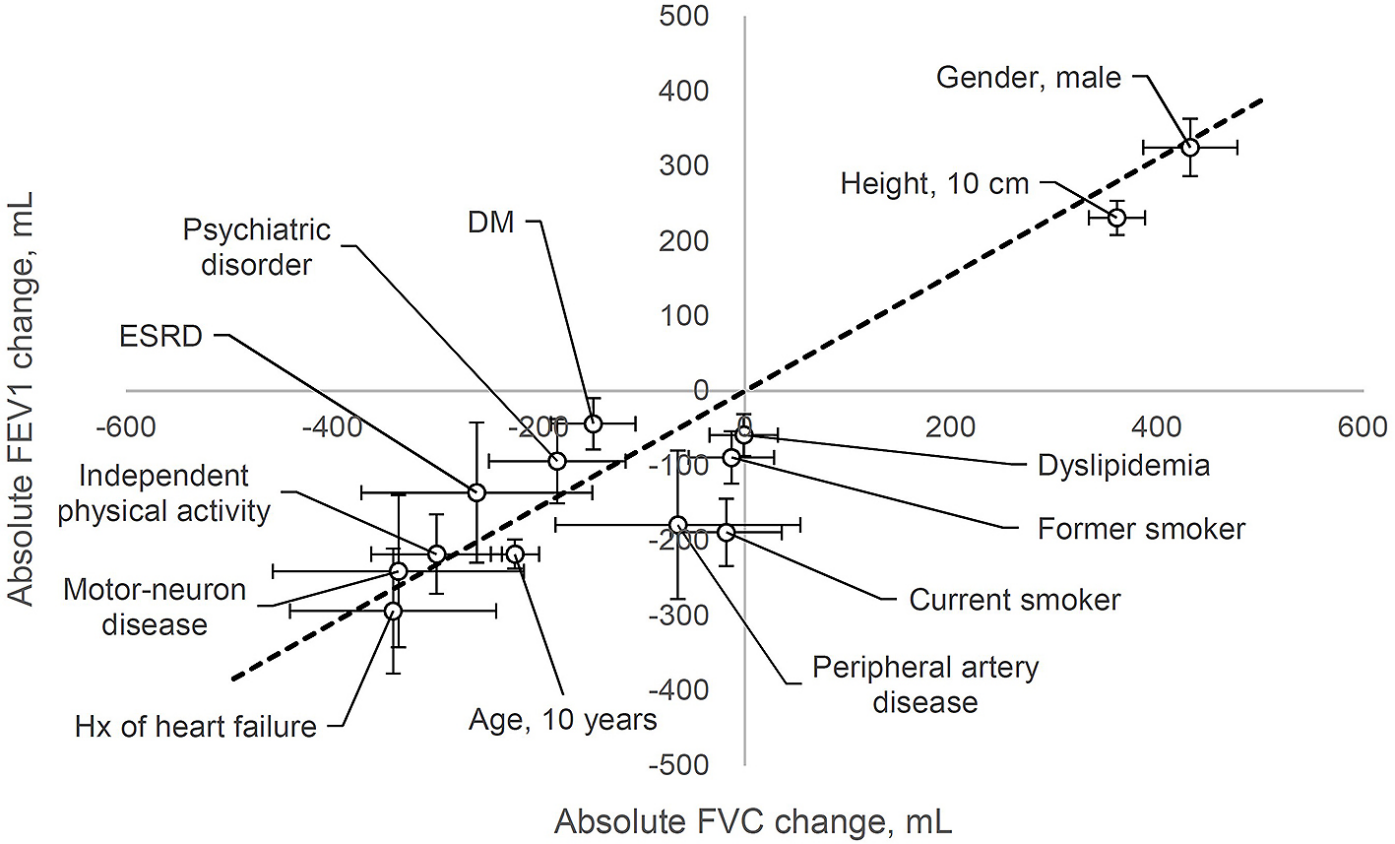
Association of age-related diseases with spirometric results. Significant parameters from Table 2 are presented in a scatter plot showing the absolute changes in forced vital capacity (FVC) on the X axis and absolute changes in forced expiratory volume in 1 second (FEV1) on the Y axis. The dotted line indicates a slope of 0.775 with a median FEV1/FVC of 77.5%. DM, diabetes mellitus; ESRD, end-stage renal disease. Bars, SD.

Data are expressed as median [interquartile range]. Multiple regression analysis (simultaneous forced entry method) was conducted to identify the factors affecting spirometric values. The dependent variables were VC, FVC, FEV1, and FEV1/FVC. In this pilot study, the independent variables included all the factors obtained by preoperative evaluation: age, height, weight, sex, smoking status, independent physical activity, hypertension, dyslipidemia, DM, ischemic heart disease, history of heart failure, peripheral artery disease, liver cirrhosis, ESRD, history of stroke, neuromuscular disease, psychiatric disorder, and malignant tumor burden. *P* < 0.05 was considered statistically significant. Since this was a pilot study for exploratory analysis, no adjustments were made to the test multiplicity. The significant factors obtained above are presented in a scatter plot, which presents the absolute change in FVC on the X axis and the absolute change in FEV1 on the Y axis (Figure 1). All statistical analyses were conducted using EZR, which is a graphical user interface for R (The R Foundation for Statistical Computing, Vienna, Austria) ^(8)^. 

## Results

### Patient characteristics

A total of 1210 respiratory function tests were conducted before general and orthopedic surgeries in patients aged ≥65 years in Itoigawa General Hospital over 5.5 years. This study excluded 14 patients due to missing data. Furthermore, 115 patients who had known respiratory diseases, 18 with chest X-ray abnormalities, 12 with pneumonectomy, and 1 who was not Japanese were also excluded. Therefore, the remaining 1050 patients were analyzed, and their characteristics are summarized in Table 1.

**Table 1.** Patients’ Characteristics in the Survey.

	Male	Female
	n = 499	n = 551
Age, years	75 [69, 81]	76 [70, 82]
Height, cm	163 [158, 167]	150 [145, 154]
Weight, kg	60 [54, 66]	50 [45, 57]
Smoking status
Never	191 (38.3)	514 (93.3)
Former	208 (41.7)	27 (4.9)
Current	100 (20.0)	10 (1.8)
Independent physical activity	28 (5.6)	43 (7.8)
Dementia	9 (1.8)	27 (4.9)
Comorbidity
Hypertension	271 (54.3)	305 (55.4)
Dyslipidemia	111 (22.2)	193 (35.0)
DM	94 (18.8)	81 (14.7)
Ischemic heart disease	48 (9.6)	11 (2.0)
History of heart failure	14 (2.8)	10 (1.8)
PAD	12 (2.4)	4 (0.7)
Liver cirrhosis	4 (0.8)	1 (0.2)
ESRD	7 (1.4)	11 (2.0)
History of stroke	56 (11.2)	35 (6.4)
Neuromuscular disease	7 (1.4)	8 (1.5)
Psychiatric disorder	20 (4.0)	35 (6.4)
Operative indication
Malignant tumor	106 (21.2)	116 (21.1)
Respiratory function
VC, L	3.25 [2.80, 3.66]	2.30 [2.00, 2.62]
FVC, L	3.11 [2.69, 3.53]	2.25 [1.94, 2.53]
FEV1, L	2.33 [1.96, 2.72]	1.76 [1.51, 2.01]
FEV1/FVC, %	75.7 [70.2, 80.1]	79.0 [74.6, 83.2]

Data are expressed as number (%) or median [interquartile range]. DM, diabetes mellitus; ESRD, end-stage renal disease; FEV1, forced expiratory volume in 1 second; FVC, forced vital capacity; PAD, peripheral artery disease; VC, vital capacity.

Patients were aged 75 [70, 81] years, and 551 (53%) were women. The number of patients who were current or past smokers was 345 (33%), and the smoking amount was described for 258 patients. The number of pack-years in current smokers was significantly higher than that in former smokers (40 [25, 50] vs 22 [10, 41]). Of the 175 patients with DM, glycemic control was measured in 155, with a median hemoglobin A1c level of 6.6% [6.1, 7.1]. Twenty-four patients had a history of heart failure in whom a chest X-ray revealed cardiomegaly with a cardiopulmonary ratio of 57% [51, 61]. Sixteen patients had neuromuscular diseases, including seven with Parkinson’s or related diseases, two with muscular dystrophy, and two with epilepsy. With regard to psychiatric disorders, 45 patients had a mood disorder, six had schizophrenia, and four had other disorders. The median values of VC, FVC, FEV1, and FEV1/FVC in all patients were 2.66 L [2.24, 3.25], 2.57 L [2.13, 3.13], 1.98 L [1.66, 2.37], and 77.5% [72.2, 81.9], respectively.

### Effect of age-related conditions and diseases on the absolute change in spirometric results

Multiple regression analysis was employed to determine the factors affecting spirometric results in older patients. After adjusting for all factors presented in Table 1, spirometric values were significantly affected by age, body height, sex, smoking status, social dependency, dyslipidemia, DM, history of heart failure, peripheral artery disease, ESRD, neuromuscular disease, and psychiatric disorders (Table 2). The factors affecting VC were the same as those affecting FVC. DM, ESRD, and psychiatric disorders were significant only for VC and FVC, but not for FEV1. Contrarily, smoking status and dyslipidemia were significant only for FEV1, but not for VC and FVC.

**Table 2.** Effect of Age-related Diseases on the Absolute Change in Spirometric Results.

	VC		FVC		FEV1		FEV1/FVC	
	VC, mL	*P*value	FVC, mL	*P*value	FEV1, mL	*P*value	FEV1/FVC, %	*P*value
Age, 10 years	–210 (–256, –165)	<0.001	–223 (–269, –177)	<0.001	–218 (–256, –179)	<0.001	–1.7 (–2.5, –0.9)	<0.001
Height, 10 cm	360 (308, 412)	<0.001	361 (308, 415)	<0.001	231 (186, 275)	<0.001	–1.7 (–2.6, –0.8)	<0.001
Weight, 10 kg	17 (–18, 53)	0.34	9 (–27, 45)	0.63	21 (–9, 52)	0.17	0.6 (0, 1.2)	0.080
Gender, male	481 (393, 569)	<0.001	432 (342, 521)	<0.001	325 (251, 400)	<0.001	–0.6 (–2.2, 0.9)	0.42
Smoking status								
Former	–14 (–94, 66)	0.73	–13 (–95, 68)	0.75	–89 (–158, –21)	0.010	–2.4 (–3.8, –1.0)	<0.001
Current	–10 (–113, 94)	0.85	–18 (–123, 88)	0.74	–189 (–278, –101)	<0.001	–5.6 (–7.4, –3.7)	<0.001
Independent physical activity	–358 (–478, –237)	<0.001	–299 (–424, –175)	<0.001	–218 (–322, –113)	<0.001	1.5 (–1.0, 3.3)	0.29
Dementia	148 (–13, 309)	0.071	71 (–93, 235)	0.40	10 (–127, 148)	0.88	–2.1 (–4.9, 0.8)	0.15
Comorbidity								
Hypertension	–25 (–85, 34)	0.40	–39 (–99, 22)	0.21	–47 (–97, 4)	0.071	–0.7 (–1.8, 0.3)	0.17
Dyslipidemia	–11 (–75, 53)	0.74	–1 (–66, 64)	0.97	–59 (–113, 0)	0.035	–1.7 (–2.9, –0.6)	0.002
DM	–159 (–238, –80)	<0.001	–147 (–228, –67)	0.003	–44 (–111, 24)	0.20	2.1 (0.7, 3.4)	0.004
Ischemic heart disease	–63 (–191, 66)	0.34	–65 (–196, 66)	0.33	–67 (–177, 42)	0.23	–1.1 (–3.4, 1.1)	0.34
History of heart failure	–331 (–524, –139)	<0.001	–341 (–537, –145)	<0.001	–294 (–458, –131)	<0.001	–1.4 (–4.8, 2.0)	0.42
PAD	–12 (–241, 217)	0.92	–65 (–298, 169)	0.53	–179 (–374, 15)	0.071	–4.3 (–8.3, –0.3)	0.034
Liver cirrhosis	–60 (–462, 341)	0.77	–122 (–531, 286)	0.56	–54 (–395, 287)	0.75	2.6 (–4.4, 9.6)	0.47
ESRD	–272 (–488, –56)	0.014	–260 (–480, –40)	0.021	–136 (–320, 47)	0.15	2.6 (–1.1, 6.4)	0.17
History of stroke	–54 (–154, 46)	0.29	–33 (–134, 69)	0.53	–24 (–109, 60)	0.57	0.5 (–1.3, 2.2)	0.58
Neuromuscular disease	–378 (–612, –143)	0.002	–336 (–537, –145)	0.006	–241 (–441, –42)	0.018	2.4 (–1.7, 6.5)	0.26
Psychiatric disorder	–280 (–404, –155)	<0.001	–182 (–312, –52)	0.006	–94 (–205, 17)	0.096	0 (–2.3, 2.3)	0.99
Operative indication								
Malignant tumor	–63 (–130, 5)	0.069	–54 (–123, 15)	0.12	–46 (–104, 12)	0.12	–0.8 (–1.9, 0.4)	0.21

Multivariate regression analyses were conducted to examine the influence of all factors presented in Table 1 on VC, FVC, FEV1, and FEV1/FVC. Data are expressed as mean (95% confidence interval). DM, diabetes mellitus; ESRD, end-stage renal disease; FEV1, forced expiratory volume in 1 second; FVC, forced vital capacity; PAD, peripheral artery disease; VC, vital capacity.

Significant parameters from Table 2 are presented in a scatter plot (Figure 1) showing the absolute changes in FVC on the X axis and absolute changes in FEV1 on the Y axis. Figure 1 shows that male sex and height were positively correlated with FVC and FEV1. Other factors, such as a history of heart failure, neuromuscular disease, and independent physical activity, were negatively correlated with FVC and FEV1 to almost the same extent as that of age.

## Discussion

This pilot study addressed the issue of the effect of age-related diseases on pulmonary function tests by secondary use of preoperative data. Multiple regression analysis revealed that in addition to well-accepted factors, such as age, height, sex, and smoking status, many age-related conditions and diseases were additively associated with decreased respiratory function. These data will provide clinically useful information for a precise interpretation of the results of pulmonary function tests in older Japanese patients. Moreover, these profiles will help estimate respiratory function in older patients in whom pulmonary function tests were not conducted before surgery.

A history of heart failure, neuromuscular disease, and independent physical activity affected the spirometric value to almost the same extent as that of age (Figure 1). Although the underlying mechanism involving heart failure is not fully understood, possible explanations include arterial stiffness, severity of mitral valve regurgitation, pulmonary vascular congestion, and enlargement of heart size ^(9), (10)^. Impaired muscle strength is one of the most frequent and persistent consequences of aging and neuromuscular disease. Respiratory muscle strength is inversely correlated with pulmonary function, and inspiratory muscle training improves respiratory muscle strength, functional capacity, lung function, and quality of life in many types of diseases ^(11), (12)^. In Parkinson’s disease, airway obstruction or restrictive pulmonary dysfunction is highly prevalent, and reduced respiratory muscle strength is observed in patients with early-stage Parkinson’s disease ^(13), (14)^. A systematic review revealed the efficacy of levodopa therapy in improving FVC in Parkinson’s disease ^(15)^.

Other factors affect respiratory function to a lesser extent than that of the abovementioned factors. Regarding peripheral artery disease, increased brachial-ankle pulse wave velocity (>1400 cm/s) is associated with moderate-to-severe airflow limitation ^(16)^. Restrictive lung dysfunction is a common complication in patients with ESRD ^(17)^. A decline in VC in patients with mood disorders is reasonable as muscle strength is inversely associated with depressive symptoms ^(18), (19)^.

A large-scale epidemiological study revealed that metabolic health is closely associated with impaired lung function ^(20)^. In the current study, patients with DM had more reduction in FEV1 than in FVC. This observation is consistent with a recent systematic review, which revealed that patients with DM had a restrictive type of lung pathology ^(21)^. Because hemoglobin A1c levels are associated with impairment of restrictive lung function, an evaluation of glycemic control is important to understand spirometric results in patients with DM ^(22)^. In the present study, patients with dyslipidemia had a greater reduction in FVC than in FEV1. The underlying mechanism for dyslipidemia inducing a reduction in FVC remains unknown. However, some reports have shown that dyslipidemia (lipoprotein (a) elevation) is associated with a reduction in FVC and FEV1 at almost the same extent ^(23)^. Further studies on the types of dyslipidemia and lipid control level are required to elucidate the effect of dyslipidemia.

The main limitation of the present study is its observational, cross-sectional design, which does not permit conclusions concerning causality. Although the exclusion criteria included known respiratory diseases, a substantial number of patients with undiagnosed chronic obstructive pulmonary disease may have been included in this survey ^(24)^. Although the amount and duration of smoking contribute to the disease severity ^(25)^, pack-years of smoking was not described in many patients. Patients who underwent non-thoracic surgery were selected for the evaluation of pulmonary function in this study, of whom 21.1% of patients had malignancy. Aging-related musculoskeletal abnormalities, such as kyphosis, might greatly affect lung mechanics ^(26)^. Although physical independence was based on the Japanese LTCI, frailty ^(27)^ and cognitive function ^(28)^ were not evaluated in this study. The severity of dementia should be quantified by mental status scales, such as the Mini-Mental State Examination ^(29)^. Biomarkers associated with a decline in pulmonary function, such as fibronectin and adiponectin, were not assessed in this study ^(30)^. Finally, this study was conducted in a single hospital and included a small number of patients.

In conclusion, this study evaluated the impact of age-related diseases on pulmonary function tests in older Japanese adults. These results will help accurately interpret respiratory function and properly manage geriatric patients under anesthesia; however, further studies are needed to confirm these findings and better understand geriatric syndrome using pulmonary function tests.

## Article Information

### Conflicts of Interest

None

### Acknowledgement

I thank Ellen Knapp, PhD, and Traci Raley, MS, ELS, from Edanz Group (www.edanzediting.com/ac) for editing the drafts of this manuscript. All analyses were reviewed by Satista Co. Ltd. (https://www.satista.jp/medical/).

### Author Contributions

MM performed the entire study.

### Ethics Approval and Consent to Participate

This retrospective cohort study was approved by the ethics committee of Itoigawa General Hospital (#2019-11) and was conducted in accordance with the principles of the Declaration of Helsinki.

### Consent for Publications

As this was a retrospective study, consent for publication was not obtained from the participants.
